# Luxation latente isolée du scaphoïde carpien chez l'enfant: à propos d'un cas

**DOI:** 10.11604/pamj.2015.20.175.6133

**Published:** 2015-02-24

**Authors:** Youssef Nader, Khalid Idrissi Koulali

**Affiliations:** 1 Pôle de Traumatologie et Orthopédie; Hôpital Militaire Avicenne, Marrakech, Maroc

**Keywords:** Dislocation, Naviculaire, enfant, latent, Dislocation, Navicular, Child, Latent

## Abstract

La luxation isolé du scaphoïde carpien est une lésion rare en particulier chez l'enfant, passant d'autant plus facilement inaperçue que le squelette du carpe est moins ossifié, dans cette observation ici rapportée, ou le diagnostic fut tardif, L'I.R.M. permet de reconnaitre la lésion, traitée par réduction chirurgicale que les auteurs considèrent essentielle même distance de la lésion.

## Introduction

La luxation du scaphoïde carpien est une lésion extrêmement rare dont les seuls cas rapportés concernent des adultes [1, 2]. Chez l'enfant, à notre connaissance, seules ont été rapportées des luxations périlunairiennes antérieures et transscaphoidiennes. Cette lésion pose un problème de diagnostic chez l'enfant jeune alors que le noyau du scaphoïde n'est pas encore ossifié. Elle pose également un problème de traitement thérapeutique, non pas tant devant une luxation fraiche que devant une atteinte ancienne comme dans notre observation.

## Patient et observation

**Figure 1:**
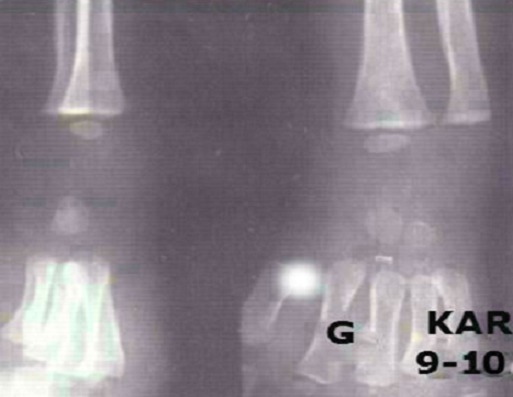
La radiographie initiale du poignet après traumatisme immediate

**Figure 2:**
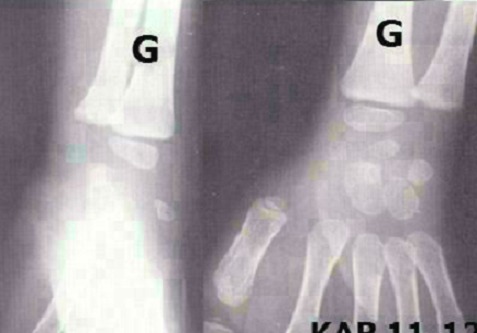
Deux ans après le traumatisme on note la présence d'un noyau d'ossification anormale du poignet gauche

**Figure 3:**
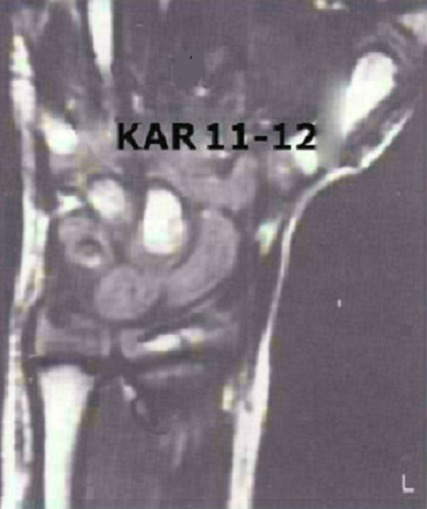
Aspect I.R.M du poignet droit, montrant le scaphoïde en place

**Figure 4:**
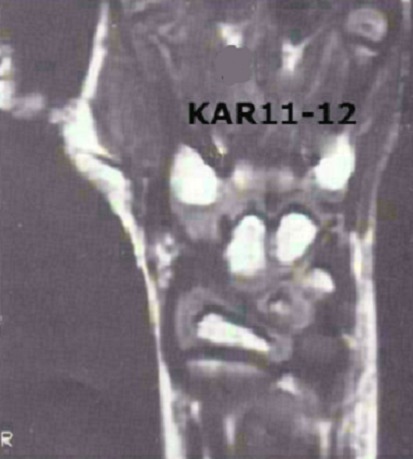
Aspect I.R.M du poignet gauche, montrant absence du scaphoïde en place et diminution de l'espace radio/grand os

**Figure 5:**
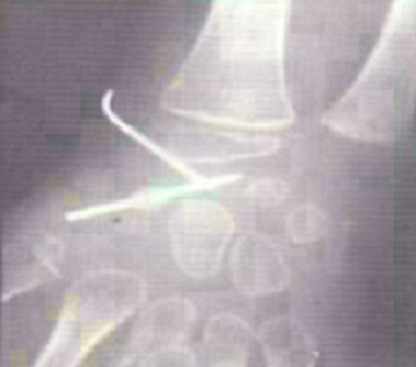
Aspect radiographique de face post opératoire après réduction chirurgicale, stabilisée par des broches radio et scaphlunaire

**Figure 6:**
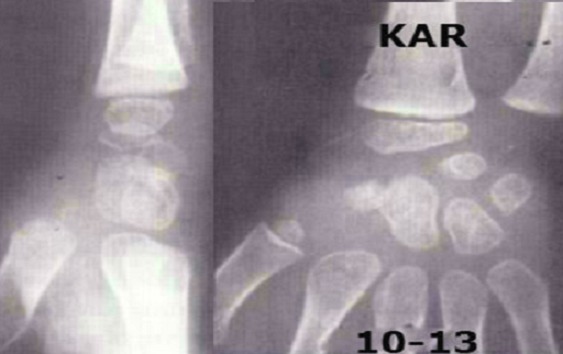
Aspect radiographique à 10 mois de recul, le pole supérieur du scaphoïde n'est pas visible, l'espace radio/grand os reste diminué

Karim. F, âgé de 03 ans victime d'un accident de la oie publique avec écrasement du carpe gauche. En urgence, on note une tuméfaction postérieure avec un gros œdème alors que l'analyse radiolographique comparative peut être considérée comme normale (Figure 1). le traitement, à cette datte, consiste en une simple immobilisation pendant une quinzaine de jours. Deux ans plus tard la maman signale que l'enfant, gêné, utilise peu sa main gauche. Cliniquement, le poignet est bien axé, mais douloureux et limité en dorsiflexion. Une tuméfaction anormale est sensible est palpé à la face postérieure du carpe. La mobilité de la colonne du pouce parait non douloureuse, alors que la force de préhension est diminuée à gauche. L'analyse radiographique comparative montre, à gauche (Figure 2). sur l'incidence de profil, la présence d'un noyau d'ossification anormal à la partie postérieure, de face, une discrète diminution de hauteur de la colonne du pouce et de l'espace radius/grand os. L'ossification du carpe est asymétrique et ne permet pas de conclure quant à l'origine de ce noyau. C'est l'I.R.M comparative des deux poignets (Figure 3) et (Figure 4) qui permettra le diagnostic de la luxation isolée du scaphoïde. Les coupes frontales montrent l'absence du scaphoïde en place à gauche. Le traitement sera chirurgical, par voie postérieure et consistera en la réintégration du pole supérieur du scaphoide. la stabilité sera assurer par deux broches, radiocarpienne et scapho-lunaire (Figure 5), avec réfection du plan ligamentaire postérieur. l'immobilisation plâtrée sera de 04 mois avec ablation des broches au deuxième mois postopératoire, l’évolution à 10 mois de recul est marquée par un pole supérieur du scaphoide n'est pas toujours visible et l'espace radio/grand os toujours diminué (Figure 6).

## Discussion

Cette observation est intéressante à double titre: d'une part en raison de sa rareté, d'autre part en raison ces problèmes thérapeutiques qu'elle souléve. la luxation du scaphoïde carpien parait être une lésion exceptionnelle chez les enfants. leur rareté s'explique par une plus grande résistance des structures cartilagineuses des os couts que les structures ossifiées [3]. Seules sont rapportés les luxations périluniennes antérieures et transcaphoidiennes [4, 5]. Dans les rares observations rapportées de l'adulte, le mécanisme associe dorsiflexion et inclinaison ulnaire du poignet occasionné par un traumatisme violent de ligament scapho-lunaire et radio-scaphoidien antérieur est constante. Seul est respecté le ligament scapho-trapézien [6] et par là, la seul vascularisation restante est celle du scaphoïde. Le diagnostic de cette lésion est difficile chez l'enfant en bas âge, au noyau scaphoidien non encore ossifié lors d'un traumatisme. Seule un examen clinique minutieux permettra, devant la présence d'une tuméfaction anormale du carpe et d'une radiographie à priori normale, de pousser les investigations plus avant et de demander une I.R.M. ultérieurement, le problème consiste à rattacher cette ossification postérieure au noyau scaphoidien. C'est l'I.R.M. comparative des poignets qui, par la qualité des images visualisant les structures non ossifiées, permettant d'analyser au mieux les différentes lésions. Chez notre jeune patient cet examen fut réalisé sous manchette plâtrée bilatérale. Le deuxième problème important posé par cette lésion est son approche thérapeutique en particulier devant une lésion ancienne. Devant une luxation fraiche, la réduction s'impose [6, 7]. Celle-ci ne sera chirurgicale qu'après échec du traitement orthopédique, posant les mêmes questions quant à la voie d'abord et la fixation qu'une lésion ancienne. Par contre en cas de lésion ancienne, comme notre observation, la question se pose d'abord de savoir s'il faut à ce stade entreprendre une chirurgie restauratrice, la résection du noyau luxé pouvant se discuter. Si un geste restaurateur est décidé, celui-ci ne pourra être que chirurgical, posant quelques questions: 1° Le choix de la voie d'abord, antérieure ou postérieure, compte tenue du risque de nécrose du noyau, scaphoidien. Il semble prudent de choisir la voie postérieure. Cette voie, parait la plus adaptée pour la réduction et la stabilisation, le risque vasculaire étant moindre que par voie antérieure dont l'intérêt est surtout de permettre La réfection du plan ligamentaire formé des ligaments radio-scaphoidien et scapho-lunaire 2° la stabilité de la réduction et son maintien compte tenu du risque de reluxation spontané après reduction. la suture du surtout fibreux postérieur, reconstruisant un semblant de plan ligamentaire. Ainsi une ostéosynthèse parait indispensable. Un brochage radiocarpienne et scapho-lunaire, permettront d'assurer la stabilité le temps de la cicatrisation, le risque d épiphysiodése intra carpienne à l'ablation des broches à deux mois postopératoire étant patent.

## Conclusion

La luxation du scaphoïde est une lésion rare, passant volontiers inaperçue chez l'enfant au noyau scaphoidien non encore ossifié. Le diagnostic lésionnel est apporté par l'I.R.M. comparative. Même à distance de la réduction chirurgicale semble s'imposer, un geste complémentaire tel qu'une arthrodèse partielle du carpe pouvant être proposée en cas d’échec par nécrose, instabilité ou arthrose.
